# Giant Adenomatoid Tumor of the Tunica Albuginea Presenting as Chronic Scrotal Swelling in an Elderly Male: A Report of a Rare Case

**DOI:** 10.7759/cureus.105326

**Published:** 2026-03-16

**Authors:** Sanhita V Patil, Suresh Phatak, Kajal Mitra, Prashant Onkar, Pranit Pantawane

**Affiliations:** 1 Radiodiagnosis, N. K. P. Salve Institute of Medical Sciences and Research Centre and Lata Mangeshkar Hospital, Nagpur, IND

**Keywords:** chronic scrotal mass, giant adenomatoid tumor, magnetic resonance imaging (mri), paratesticular tumor, scrotal ultrasound

## Abstract

Paratesticular tumors are uncommon intrascrotal neoplasms involving a heterogeneous group of benign and malignant lesions. Adenomatoid tumor accounts for only 1-2% of all scrotal tumors. Although adenomatoid tumors are the most prevalent benign neoplasms of the paratesticular region and commonly arise from the epididymis, primary involvement of the tunica albuginea remains rare. Such an unusual imaging presentation might resemble that of malignant tumors (like liposarcoma, leiomyosarcoma, and rhabdomyosarcoma), posing a diagnostic challenge.

We report a 60-year-old male with a 15-year history of scrotal swelling and recent onset of mild dragging and low-grade pain. Subsequent scrotal ultrasound and contrast-enhanced MRI were performed, followed by surgery and histopathological examination (HPE), which confirmed an adenomatoid tumor arising from the tunica albuginea. Unlike the typically small lesions seen in younger adults, this case involved a giant adenomatoid tumor arising from the tunica albuginea in an older patient, highlighting its unusual presentation.

## Introduction

Paratesticular tumors account for approximately 7-10% of all intrascrotal masses [1]. These tumors arise from structures including the epididymis, the spermatic cord containing the vas deferens, the pampiniform plexus, vessels, and nerves, as well as the tunica vaginalis and tunica albuginea and supporting connective tissues [1]. Among benign paratesticular neoplasms, adenomatoid tumors are the most common and are believed to originate from mesothelial cells [1,2]. Adenomatoid tumors typically present as slow-growing, painless scrotal masses in middle-aged men; however, cases mimicking malignant testicular tumors have been reported, complicating clinical decision-making [2,3]. Rare presentations have also been described in pediatric patients and in unusual anatomical locations such as the tunica albuginea, underscoring their diverse spectrum [4].

Ultrasonography (US) is the first-line imaging modality for evaluating scrotal masses, aiding in differentiating between intratesticular and extratesticular lesions [5]. Magnetic resonance imaging (MRI) plays a complementary role in further characterization, assessment of lesion margins, and tissue composition when sonographic findings are indeterminate [6]. Despite advances in imaging, definitive distinction between benign and malignant paratesticular tumors remains challenging, often necessitating surgical excision and HPE confirmation [7].

We present this case of a giant adenomatoid tumor of the tunica albuginea in an elderly male with multimodality imaging correlation and histopathological confirmation to highlight the rare occurrence of this tumor at this site, particularly with giant size and in an elderly patient, and to emphasize the importance of recognizing its imaging features, as it may mimic malignant paratesticular tumors and pose a diagnostic challenge.

## Case presentation

**Figure 1:**
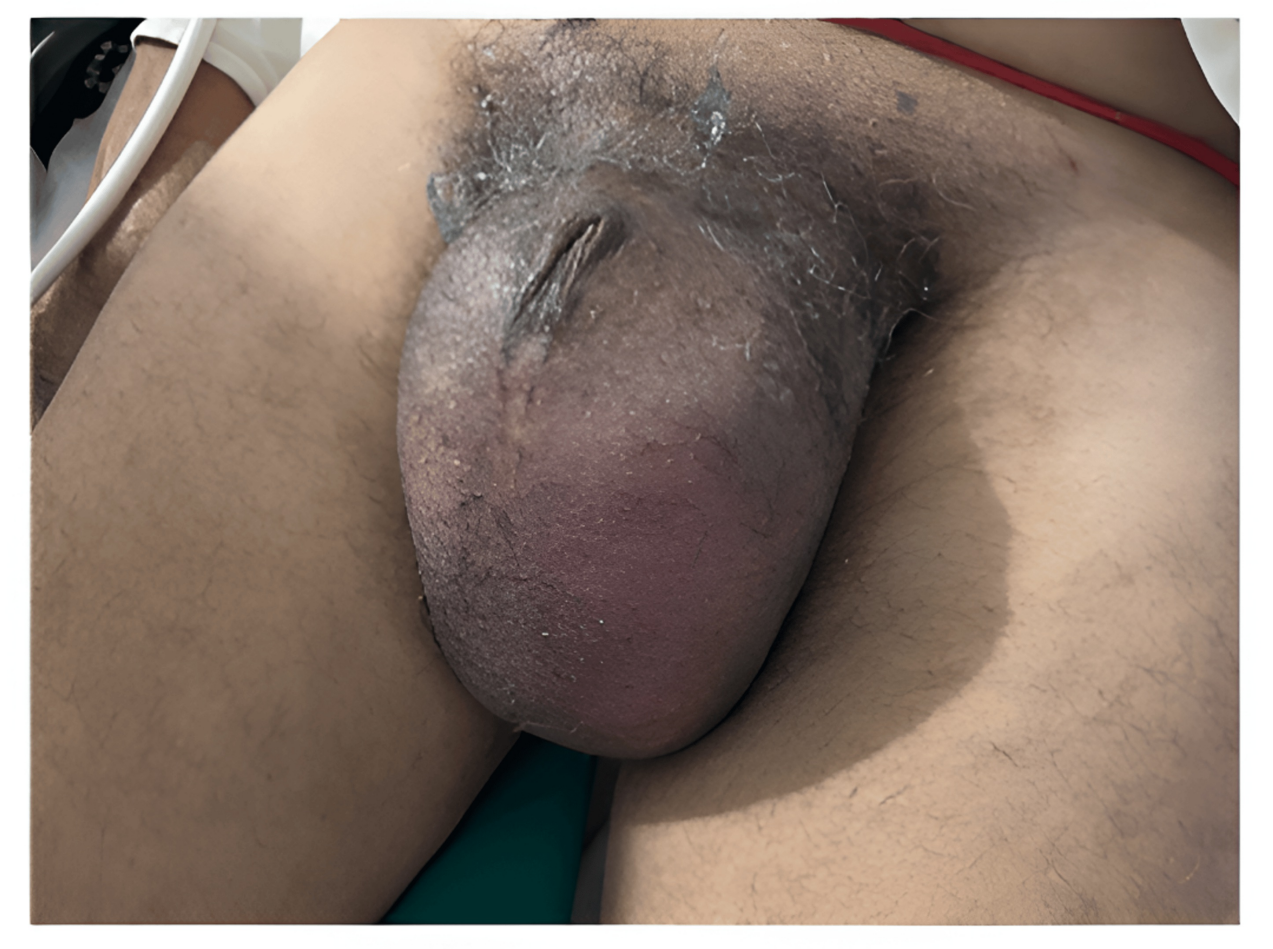
Clinical photograph of the patient at the presentation The image shows left hemiscrotal swelling with mild overlying erythema.

A 60-year-old male presented for the first time to the surgery outpatient department with left-sided scrotal swelling for 15 years. The swelling had remained asymptomatic and stable in size until five days prior to presentation, when he developed mild dragging discomfort and low-grade pain. There was no history of trauma, fever, urinary symptoms, or systemic illness. On clinical examination, a firm, mildly tender mass was palpable in the left hemiscrotum. The testis appeared displaced but clinically inseparable. Overlying skin demonstrated mild erythema, with no associated ulceration or discharge (Figure 1). The patient was referred for scrotal ultrasonography for further evaluation.

**Figure 2:**
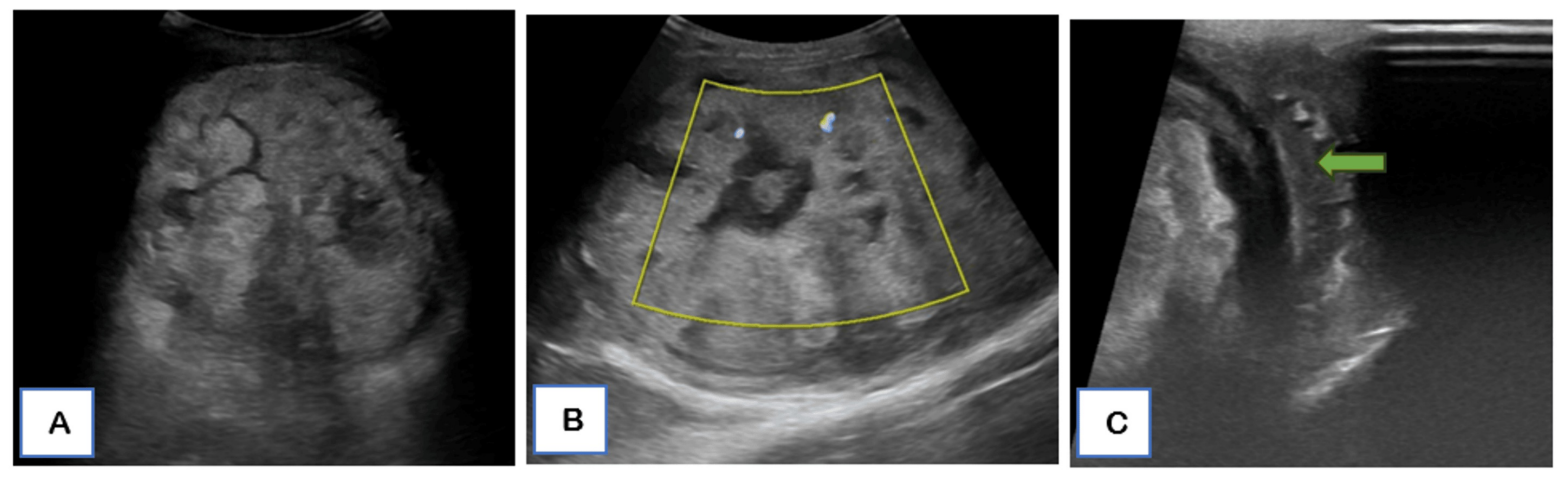
Scrotal ultrasound images (A) A well-defined, heterogeneous, predominantly hyperechoic lesion was seen in the left scrotum. (B) On color Doppler, it showed minimal vascularity. (C) The left testis was visualized separately; however, it was significantly compressed (green arrow).

Scrotal ultrasound demonstrated a large heterogeneous predominantly hyperechoic lesion in the scrotal sac on the left side with minimal internal vascularity on color Doppler imaging. The left testis was compressed but distinct from the lesion, suggesting extratesticular origin (Figures 2A-2C). The left epididymis and spermatic cord appeared normal in morphology.

**Figure 3:**
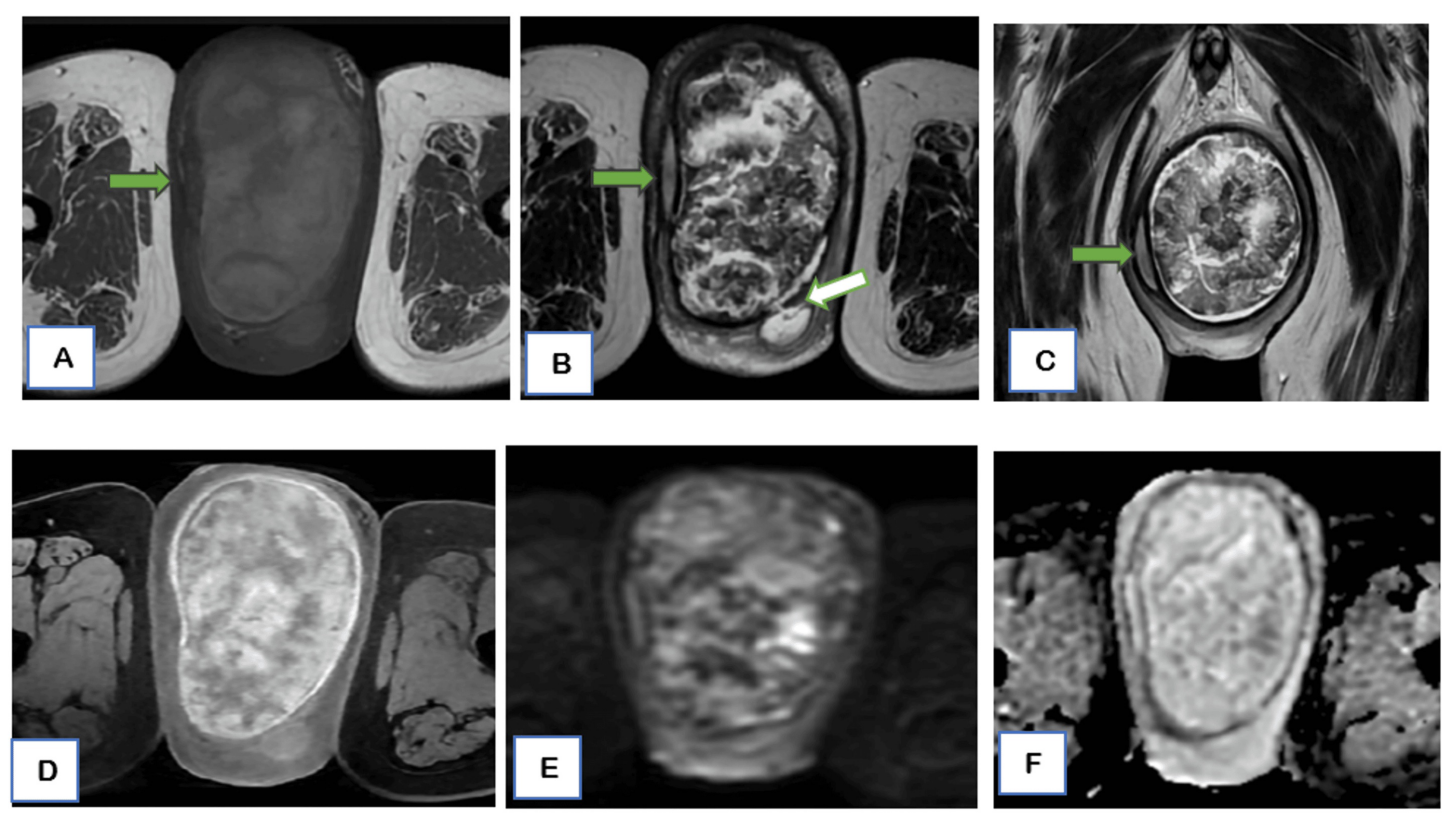
Scrotal MRI images A large, well-defined, encapsulated heterogeneous lesion was seen, which showed solid-cystic components on both axial T1WI (A) and T2WI (B, C) sequences. The left testis is seen compressed and displaced laterally to the right, with maintained fat planes (green arrow). (B) On axial T2WI image, a small capsular focal defect was seen with fluid seen leaking and forming a small collection in the scrotal wall (white arrow).  (D) On dynamic post-contrast axial images, early avid heterogeneous enhancement of the solid areas, with internal non-enhancing regions corresponding to cystic or degenerative components was noted. (E) diffusion weighted imaging (DWI) and (F) apparent diffusion coefficient (ADC) – revealed few small areas of mild diffusion restriction within solid components.

Subsequent MRI of the scrotum demonstrated a large, well-defined, encapsulated solid-cystic mass measuring approximately 10.4 x 8.3 x 14.2 cm (cranio-caudal (CC) X traction (TR) X anteroposterior (AP)) within the left scrotal sac. The lesion displaced the left testis right-laterally and appeared to originate from the paratesticular region, closely related to the tunica albuginea. The testicular parenchyma was compressed but remained distinct, with preserved surrounding fat planes and no evidence of direct invasion. The left epididymis was visualized separately and appeared slightly compressed by the lesion. The left spermatic cord was normal in morphology. On T1-weighted, T2-weighted, and short tau inversion recovery (STIR) sequences, the mass showed heterogeneous signal intensity with both solid and cystic components. No macroscopic fat component was identified. Diffusion-weighted imaging revealed few areas of mild diffusion restriction within the solid components (apparent diffusion coefficient (ADC) value of 1.2 ×10⁻³ mm²/s). Post-contrast images demonstrated early, heterogeneous avid enhancement of the solid areas, with internal non-enhancing regions corresponding to cystic or degenerative components. A focal capsular defect was seen with peripherally enhancing fluid tracking along the lateral and posterior margins into the scrotal wall, likely representing inflammatory leakage or partial rupture and correlating with the recent onset of pain and erythema (Figures 3A-3F). These features favored a benign neoplasm, likely a giant adenomatoid tumor arising from the tunica albuginea, which was confirmed later.

**Figure 4:**
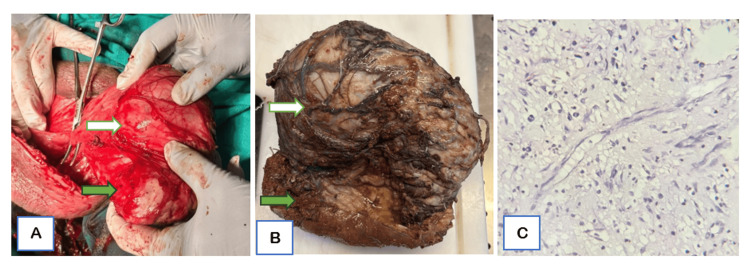
Post-operative clinical and histopathology images (A) Intraoperative photograph demonstrating a large paratesticular mass (white arrow) in tunical coverings with a separate left testis (green arrow). (B) Gross examination of the excised specimen shows the tumor closely related to the tunica albuginea (white arrow) and the separate left testis (green arrow). (C) Histopathology (H&E stain) reveals characteristic cells with vacuolated cytoplasm and few scattered inflammatory cells.

Pre-operative serum tumor markers, including alpha-fetoprotein (AFP), beta-human chorionic gonadotropin (β-hCG), and lactate dehydrogenase (LDH), were within normal limits, with AFP 2.04 IU/mL, β-hCG <2 IU/L, and LDH 123 U/L. Thereafter, the patient underwent surgical exploration. Intraoperatively, a large, well-circumscribed paratesticular mass arising from the tunica albuginea was identified in close relation to the testis. The left epididymis and spermatic cord were identified separately and appeared grossly normal. A testis-sparing mass excision was initially planned; however, considering the lesion’s size and involvement, a left-sided orchiectomy with complete excision of the mass was performed (Figure 4A), and the specimen was sent for histopathological examination. On histopathological examination (HPE), gross assessment showed a capsulated mass with regular external contours (Figure 4B). Microscopic evaluation revealed adenoid patterns composed of cells with clear cytoplasm and scattered inflammatory cells, without significant cytologic atypia or increased mitotic activity (Figure 4C). These features were consistent with an adenomatoid tumor.

## Discussion

Paratesticular masses encompass a wide pathological spectrum, including benign and malignant mesenchymal and mesothelial tumors [1]. Paratesticular adenomatoid tumor is the most common benign paratesticular neoplasm [1,3]. The epididymis represents the most frequent site of origin of paratesticular adenomatoid tumors, accounting for approximately 70% of cases, with the tunica vaginalis being the next most common location. In contrast, involvement of the tunica albuginea is distinctly uncommon [1,8].

Clinically, adenomatoid tumors most commonly present in the third to fifth decades of life, though cases have been reported across a wide age range. Patients usually present with a slow-growing, painless scrotal swelling, which is often incidentally detected; acute pain is uncommon and may occur secondary to inflammation or secondary changes [1,7]. Ultrasound and MRI are the primary imaging modalities for evaluation of paratesticular masses; CT has a limited role and is generally not routinely indicated and is reserved for staging when there is suspicion for malignancy [1].

On ultrasound, adenomatoid tumors typically appear as well-circumscribed extratesticular solid masses, commonly arising from the epididymis or tunical coverings. They may be hypoechoic, isoechoic, or mildly heterogeneous relative to the adjacent testis. Posterior acoustic enhancement is uncommon, and calcification is rare. Color Doppler evaluation generally demonstrates mild internal vascularity, helping differentiate them from avascular cystic lesions, though the vascular pattern is not specific [5].

MRI is particularly valuable in confirming the extratesticular origin, defining the relationship of the lesion to the tunica albuginea and testicular parenchyma, and assessing internal composition. On MRI, these tumors are typically well-defined and capsulated, iso- to mildly hypointense on T1-weighted images, and show variable signal intensity on T2-weighted sequences depending on stromal content or cystic degeneration. Post-contrast sequences typically demonstrate early heterogeneous enhancement, while diffusion-weighted imaging may show mild restriction in solid cellular areas. Contrast-enhanced MRI further aids in better delineation of lesion margins and internal enhancement characteristics, helping differentiate benign paratesticular masses from malignant testicular tumors [6]. The presence of tubular and angiomatoid architectural patterns composed of bland mesothelial cells with eosinophilic or vacuolated cytoplasm on histopathology, along with immunohistochemical positivity for mesothelial markers such as calretinin, cytokeratin, and WT-1, supports the diagnosis of an adenomatoid tumor [5].

The following represent the key imaging differentials for a paratesticular mass: 1. Lipoma: the most common benign paratesticular tumor demonstrates homogeneous fat signal intensity on MRI with suppression on fat-saturated sequences [1,8]; 2. Leiomyoma: a benign smooth muscle tumor presenting as a well-defined solid mass with homogeneous enhancement [1]; 3. Fibrous pseudotumor: a reactive fibroinflammatory lesion often associated with prior trauma or inflammation and characteristically shows very low signal intensity on T2-weighted MRI due to dense fibrosis [1,9]; 4. Papillary cystadenoma: often associated with von Hippel-Lindau disease, presents as a cystic lesion with internal papillary projections [1].

Adenomatoid tumors are benign mesothelial neoplasms, and complete surgical excision is the treatment of choice, usually performed as local excision or testis-sparing surgery when feasible. However, in cases where the lesion is large or closely associated with the testis and malignancy cannot be excluded preoperatively, orchiectomy may be performed [1,8]. The prognosis is excellent, as these tumors demonstrate benign behavior with no reported malignant transformation or recurrence following complete excision [5].

In the present case, the patient had an unusually long clinical history of approximately 15 years and was in the sixth decade of life, older than the typical age group reported for this tumor. Imaging demonstrated a large, well-defined encapsulated paratesticular mass arising from the tunica albuginea with solid-cystic components, causing displacement and marked compression of the adjacent testis. Heterogeneous enhancement and mild diffusion restriction raised suspicion for malignancy, prompting surgical exploration. Intraoperatively, the mass was found to arise from the tunica albuginea with severe compression of the testis, making testis-sparing excision impractical and necessitating orchiectomy. Histopathological examination confirmed the diagnosis of an adenomatoid tumor, underscoring this case as an unusual presentation in terms of size, site of origin, and patient age.

## Conclusions

Paratesticular tumors can be difficult to characterize because benign and malignant lesions may show similar imaging appearances. Although ultrasound and MRI are valuable for lesion localization and structural assessment, histopathological evaluation remains essential for definitive diagnosis. This case illustrates an uncommon presentation of a large adenomatoid tumor arising from the tunica albuginea in an elderly patient, emphasizing the need for careful clinicoradiological correlation to ensure accurate diagnosis and appropriate surgical management.
